# Diagnosing a 12-Item Dataset of Raven Matrices: With Dexter

**DOI:** 10.3390/jintelligence8020021

**Published:** 2020-05-06

**Authors:** Ivailo Partchev

**Affiliations:** Cito, 6814 CM Arnhem, The Netherlands; Ivailo.Partchev@cito.nl

**Keywords:** intelligence tests, classical test theory, IRT, interaction model, test-item regression

## Abstract

We analyze a 12-item version of Raven’s Standard Progressive Matrices test, traditionally scored with the sum score. We discuss some important differences between assessment in practice and psychometric modelling. We demonstrate some advanced diagnostic tools in the freely available R package, dexter. We find that the first item in the test functions badly—at a guess, because the subjects were not given exercise items before the live test.

## 1. Introduction

Myszkowski and Storme (2018) have applied a number of binary and polytomous item-response theory (IRT) Lord (1980) models to the last series of Raven’s Standard Progressive Matrices (SPM) test Raven (1941), further referred to as the SPM-LS test. They have made their dataset publicly available, and the *Journal of Intelligence* has proposed a special issue where other researchers are encouraged to present their own analyses.

The idea is not entirely new. Back in 1976, Thissen (1976) tried to apply Bock’s nominal response model Bock (1972) to Raven’s matrices as an attempt to throw light on the functioning of the distractors and improve scoring in the lower ability range. It is easy to overlook this publication as it came so incredibly early, some five years before Bock and Aitkin (1981) proposed a really practicable way to estimate the model.

To start with the big question of whether applying complex IRT models to an old, venerable test of intelligence should be an improvement: I have not one but two answers. One is “possibly”, the other “certainly not”. The duplicity arises from the fact that it is not possible to have methods and criteria that would be equally appropriate to summative assessment, formative assessment, survey research, methodological research, or substantive research.

Consider assessment. Computer-assisted learning has developed at staggering rates, becoming essentially intertwined with formative assessment. Operating within the effort to increase ability, we can even enjoy the luxury of being able to ask the same item multiple times and observe learning happen. Summative assessment has remained more traditional: We tend to interrupt the learning process for a while, hoping that ability will remain unchanged during testing, and praying that the items have not been compromised by disclosure. The two modes are not simply different—they are more like opposites. Hence, there is no methodological one-size-fits-all—not even within assessment practice.

On the other hand, not everybody who analyzes test data is busy grading exams. Some might be studying populations, as is the case with PISA, TIMSS and friends. Others might be interested in the way people behave when answering educational or intelligence tests. They will come up with ideas and hypotheses whose evidential support will have to be demonstrated, since statements are not limited to a specific individual or projected to a specific finite population but generalized beyond. Goodness of fit plays a very different role in such circumstances than in the more artisanal job of making a measurement instrument for testing.

In the role of researchers, we might for example ask whether persons are guessing responses at random, and we can try to formalize the question into a testable model. It is a perfectly valid discussion Azevedo (2009); Glas (2009); Maris and Bechger (2009); Partchev (2009); San Martín et al. (2009); Thissen (2009); von Davier (2009) whether such a model, say the 3PL, is a good idea from a substantive or mathematical point of view. From my participation in that dispute it is clear that I am not very enthusiastic; see also Appendix B for some results in applying the 3PL model on the SPM-LS dataset. However, this is not the same as porting the 3PL model into assessment practice, the latter being predominantly ruled by the sum score. This is mainly for two reasons: (i) The sum score makes sense in a particular social situation and (ii) it seems to capture most of the essential information in the responses.

As Dorans (2012) notes, commenting on earlier work by Paul Holland, test takers can assume multiple roles: Those of learners, examinees, or contestants. Quoting from his abstract: “Test takers who are contestants in high-stakes settings want reliable outcomes obtained via acceptable scoring of tests administered under clear rules.” Telescoping to sports, where fairness is also a major issue, the 2020 edition of the ATP rulebook ATP Tour Inc. (2020) defines every conceivable rule and situation in the game of tennis on 374 pages (beats the APA Publication Manual American Psychological Association (2010) by more than 100 pages). Nothing is left to chance, everything is specified well before the game starts, and just how bizarre the idea that the scoring rules might be defined post hoc, based on a fairly opaque analysis of the results, and placidly assuming that athletes cheat as a rule. However, this is exactly what the 3PL model proposes.

Similar objections may be raised against the idea to ’exploit’ the potentially useful information in the wrong responses by fitting a nominal response model. Investigate in research—yes; exploit in assessment—rather not. When we are to pass judgement over individuals, our thinking tends to be more binary: Either the distractors are wrong and should get no credit, or they are sensible and should get partial credit. In either case, it should be part of the rules before the referee shouts “Time!”.

The need for simple scoring rules that are known before testing has begun, are easily explained to all parties involved, and are widely accepted as fair, is one of the main reasons why most assessment programs tend to rely on the sum score. When the test has more than one form, the choice is mainly between classical test theory (CTT) and equipercentile or kernel equating (still a hot topic, to judge by the number of recent books González and Wiberg 2017; Kolen and Brennan 2014; von Davier 2011; von Davier et al. 2004), or IRT, which provides an alternative solution to the equating problem. However, we would be interested primarily in models with sufficient statistics, such as the Rasch or the partial credit model, because they preserve the scoring rule (in the case of one test form, the ability estimates are just a monotone transformation of the sum score).

Another important advantage is that the degree of misfit of the IRT model would indicate the extent to which our scoring rule misses out potentially useful information. This is more realistic on the item level, where it can be a valuable tool in quality assurance. At test level and within IRT, it is more difficult to demonstrate misfit in practice (see also Appendix C). The search for that important thing that is not already captured by the sum score has become something of a Holy Grail in psychometrics—since the day when they added a second parameter to the Rasch model and up to the latest advances in cognitive diagnostic assessment Leighton and Gierl (2007). I have followed with great interest, have often been disappointed, and will probably be just as enthusiastic when the next wave appears.

What follows is an example of the initial data crunching that would be done at an educational testing institute when the data from a new test comes in. A careful exploratory analysis should always precede whatever comes next, whether assessment or further modelling and research; and we should not forget that the properties of an instrument and the properties of a dataset collected with it are not the same thing.

While playing Sherlock Holmes with the SPM-LS data, I take the opportunity to present our freely available R package, dexter, Maris et al. (2019) because it has been developed especially for this purpose and combines traditional and novel methods. The accent is on assessing and understanding item fit. There is no attempt at an exhaustive analysis of the psychometric properties of the 12-item test form, SPM-LS. Raven’s matrices have been around for about 80 years and much is known about them—for example, Brouwers et al. (2009) examine 798 applications in 45 countries (N = 244,316) published between 1944 and 2003. Besides, an insight into the properties of the short form can be seen as the collective endeavour of the whole special issue—see, for example, Garcia-Garzon et al. (2019) for a factor-analytic analysis that shows the SPM-LS to be essentially unidimensional.

## 2. Materials and Methods

### 2.1. Data

The data is as supplied with the original study by Myszkowski and Storme (2018): The responses of 499 French undergraduate students aged between 19 and 24 to the twelve items of SPM-LS.

### 2.2. Methods

All analyses have been performed with dexter Maris et al. (2019), a freely available package for R Core Team (2013). Dexter has been created to be as useful as possible to both researchers and test practitioners, as long as they stay with models that have sufficient statistics for their parameters Andersen (1973). Every dexter project starts, as appropriate for testing, with a complete enumeration of the scoring rules for each item: Every admissible response gets mapped to an integer, with 0 as the lowest item score. Out of these rules, the program creates automatically a state-of-the-art relational data base optimized for the typical structure of test data.

The toolbox for assessing the quality of the items includes, among others:

- the usual statistics of classical test theory (CTT) Lord and Novick (1968);
- distractor plots, i.e., nonparametric regressions of each response alternative on the sum score;
- item-total regressions obtained directly from the data, from the calibration model (Rasch or partial credit), and from Haberman’s interaction model Haberman (2007).

There is a companion package, dextergui Koops et al. (2019), providing an easy graphical user interface (GUI) to the basic functions. The GUI is very convenient: All tables are live, they can be sorted on each column, and clicking anywhere on the table opens up the appropriate graphs. However, in a paper like this it is easier to reproduce a script (see Appendix A) than to explain a GUI.

Readers of this journal will hardly need CTT statistics like item facility and item-total correlation, the Rasch model Rasch [1960] (1980), or the partial credit model (PCM) Masters (1982) explained. What we call distractor plots are non-parametric regressions of response alternatives on the total score. We produce them by estimating the density of the total score, overall and for each response alternative, and applying Bayes’ rule to obtain the density of each response alternative given the total score.

**Figure 1:**
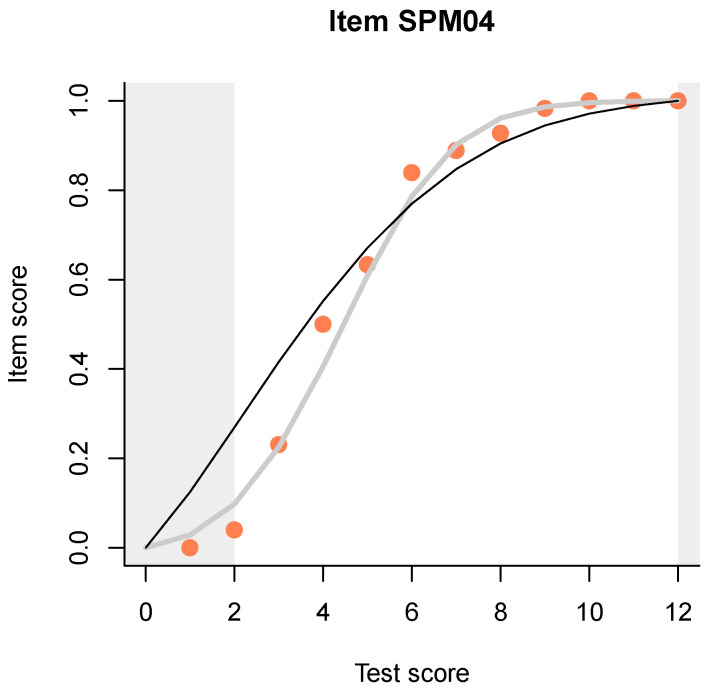
Example plot comparing three item-total regressions for the fourth item. Pink dots show the observed regression (in this case, proportion of correct responses at each distinct total score), predictions from the Rasch model are shown with a thin black line, and those from the interaction model with a thick gray line.

A useful and novel method is a plot (example shown in Figure 1) that compares three item-total regressions:

- the empirical regression, shown with pink dots and representing, simply, the proportion of correct responses to the item (or the mean item score, for partial credit items), at each test score;
- the regression predicted by the Rasch (or partial credit) model, shown as a thin black line;
- the regression predicted by Haberman’s interaction model, shown as a thicker gray line.

Item-total regressions (ITR) are somewhat similar to item response functions (IRF), but there are some important differences. The IRF combines an unobservable quantity on an arbitrary scale (on the *x* scale) with observable data (on the *y* axis) while the ITR only involves observable data.

What, however, is the interaction model? Well hidden in a book on an entirely different subject, Haberman’s interaction model Haberman (2007) remains relatively unpopular and underestimated. We (the developers of dexter) have found it to be a very useful diagnostic tool, and we have generalized it to also handle polytomous items. The interaction model can be seen equivalently as a model for locally dependent items, a Rasch model where difficulty depends on item and score, and an exponential family model for classical test theory, as can be seen from the following equations:

(1) $$P\left(x|\theta \right)\propto \exp(\theta x_{+}-\sum_{i}\beta_{i}x_{i}+\sum_{i}\sum_{j>i}\left(\sigma_{i}+\sigma_{j}\right)x_{i}x_{j})$$

(2) $$P\left(x|\theta \right)\propto \exp(\theta x_{+}-\sum_{i}\left(\beta_{i}+\sigma_{i}x_{+}\right)x_{i})$$

(3) $$P\left(X|\theta \right)\propto \exp\left(\sum_{i}\beta_{i}x_{+i}+\sum_{i}\sigma_{i}\sum_{p}x_{pi}x_{p+}+\sum_{s}n_{s}\ln\lambda_{s}\right)$$

where *i* and *j* index items, *p* indexes persons, *s* indexes sum scores, and + stands for summation. *x* are observed item responses, x a response vector, and X a matrix of responses. θ are latent abilities, β item difficulties, and σ are the item-specific interaction parameters featured in Haberman’s model. The λ are there to reproduce (i.e., perfectly fit) the score distribution, and may be called score-parameters.

Each of these three representations can serve as the point of departure for a potentially useful discussion. Because our interest here is mainly in item fit, we will concentrate on the third one. We observe that the three terms in the exponential ensure that the model will reproduce perfectly, through the three sets of parameters, β, σ, and λ, the classical item facilities, the correlations of the item scores with the total score, and the distribution of the total scores. Note that this is more or less everything that we want to know about the data within CTT.

Let us return to Figure 1. I have deliberately chosen the item that deviates the most from the Rasch model in having a higher discrimination. The IM readily detects that, in fact, the 2PL model can be shown to be a low-rank approximation to the IM, so we have even more flexibility with the IM than with the 2PL model. However, unlike the two-, three- or many-PL models, the IM has sufficient statistics, it can be estimated via the conditional likelihood, and it makes predictions conditional on the observed total score, not on a hypothesized, latent quantity. This makes it much more appropriate for evaluating item fit.

Observe how, when the Rasch model and the IM deviate for an item, the pink dots representing the empirical item-total regression tend to cluster around the IM curve. This is what one typically sees in practice, and the points tend to get closer to the line as the sample size increases. In other words, not only does the IM reproduce exactly the three most interesting aspects of the response data from a CTT point of view, but it seems to capture all systematic deviations from the Rasch model, leaving out just the random noise. To make the plots even more legible, we have introduced ‘curtains’ slightly obscuring but not hiding the lower and upper 5% of the data as measured on the test score. This helps concentrate on the really important differences among the three ITR.

Neither the Rasch model nor the IM make any provisions for random guessing. The 3PL model, on the contrary, assumes that people always guess, and then tries to fit a curve with a particular shape to the data. Even if that is successful (the model has an identification problem, as shown in Azevedo 2009; Glas 2009; Maris and Bechger 2009; Partchev 2009; San Martín et al. 2009; Thissen 2009; von Davier 2009), the data can lie near to the curve for many reasons, one of which is random guessing. None of the three models have a device to tell us whether people are actually guessing or not.

The two smoothed ITR start and end at the same points as the observed ITR. Inevitably, both the observed and the predicted item score must be 0 when the total score is 0, and when a person achieves a full total score, the item score for each item must also take the maximum possible value. This gives a specific aspect to the ITR depending on the slope. When an item discriminates better than predicted by the Rasch model, the ITR of the IM retains the same sigmoid shape but gets steeper. When discrimination is low, typical of badly written items, the curve starts to bend, resembling a cubic polynomial. This is particularly expressive when the ITR must accommodate a negative slope in the middle, typical of items with a wrong answer key. When the slope is small or negative, the ITR of the IM suggests that persons of low ability (say, at the left curtain) have a spuriously high probability of a correct response. This is not necessarily due to guessing.

To summarize: I believe that items discriminating similar to or better than what is expected under the Rasch model can be used without consternation: Differences in the slope will cancel when we sum together even a very modest number of item scores (see also Appendix C). Low discrimination always means trouble of one kind or another. So, my recommended workflow is to catch such items, starting with the item-total and item-rest correlations and proceeding with the item-total regressions. A careful analysis of the distractor plots for the offending items will help diagnose what is wrong with the item and suggest a revision.

## 3. Results

The 12-item test, SPM-LS, has a decent Cronbach alpha of 0.81, and an average item facility of 0.65. The average correlation with the total score (rit) is 0.57, and the average correlation with the rest score (rir) is 0.47.

Table 1 shows the essential item level CTT statistics. As expected from the structure of the SPM test, the item facilities progressively decrease with the notable exception of the first item. Discrimination, as characterized by the rit and the rir, is highest in the middle and lowest at both ends, which is what one would expect from point-biserial correlations. However, the discrimination for the first item is a bit too low, especially as the item does not appear to be as easy as anticipated.

**Figure 2:**
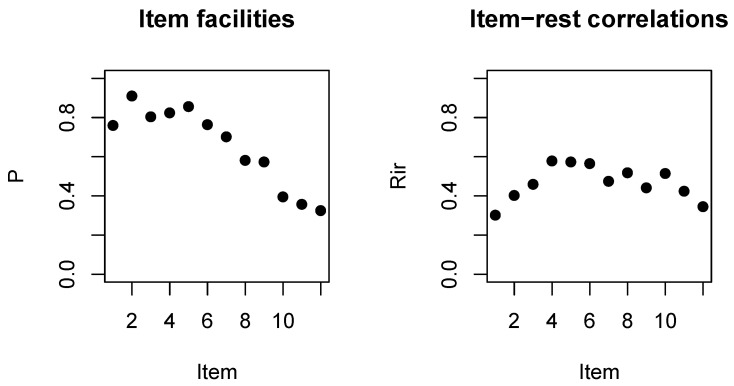
Item facility (**left**) and correlation with the rest score (**right**) by position of the item in the SPM-LS test.

These observations are facilitated by the plots on Figure 2.

**Figure 3:**
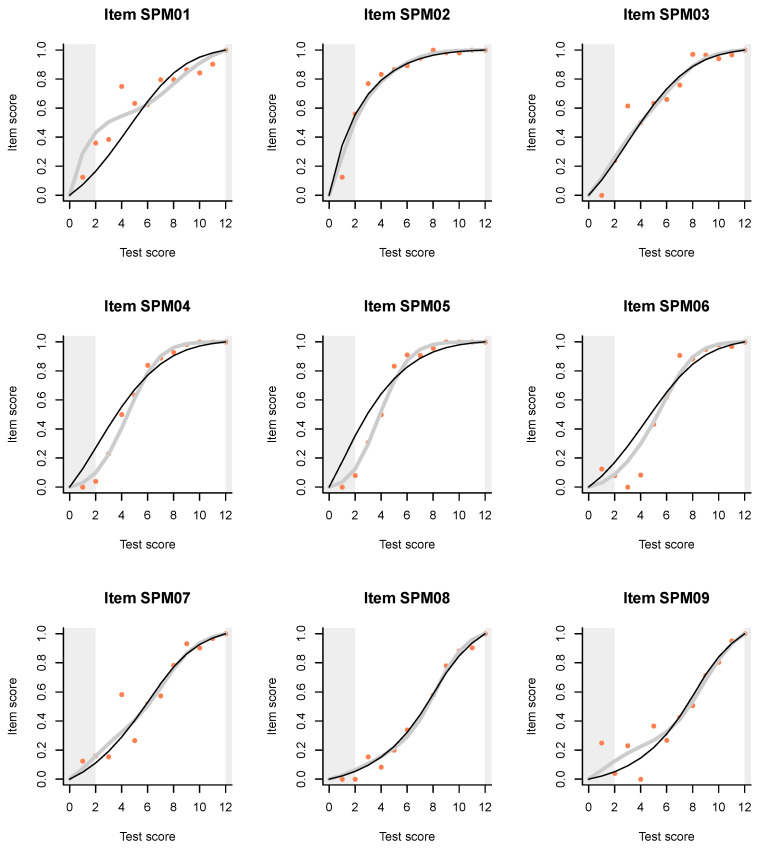

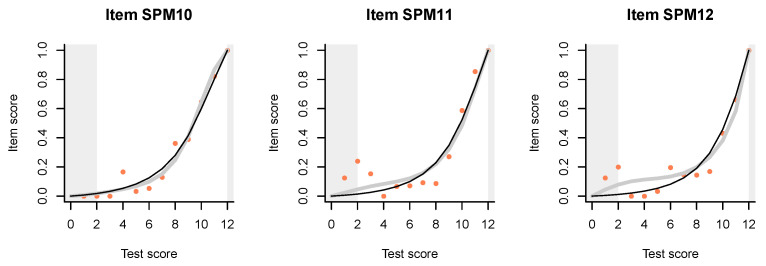
Item-total regressions for the items in the SPM-LS test obtained from the data (pink dots), the Rasch model (thin black lines), and the interaction model (thick gray lines).

The slight hint that the first item, SPM01, may be out of line with the others, becomes quite dramatic when we examine the ITR plots (Figure 3). Just compare the plots for the first two items, which are supposed to be very similar. For item SPL02, the Rasch model and the IM agree almost perfectly. According to both models, persons of low ability, say at the left curtain (fifth percentile) have a high probability, well over 0.5, to answer correctly, but this is obviously because the item is very easy.

The plot for item SPM01 is very different. The IM curve has a very particular and irregular shape, thanks to which it deviates sharply from the Rasch model in the lower ability range, but much less in the upper range. What is going on among persons of lower ability? Are they guessing? The pseudo-guessing parameter in the 3PL model (Appendix B) is equal to zero for both items, and what is the logic to guess when the item is so easy? Why on the first item but not on the second?

**Figure 4:**
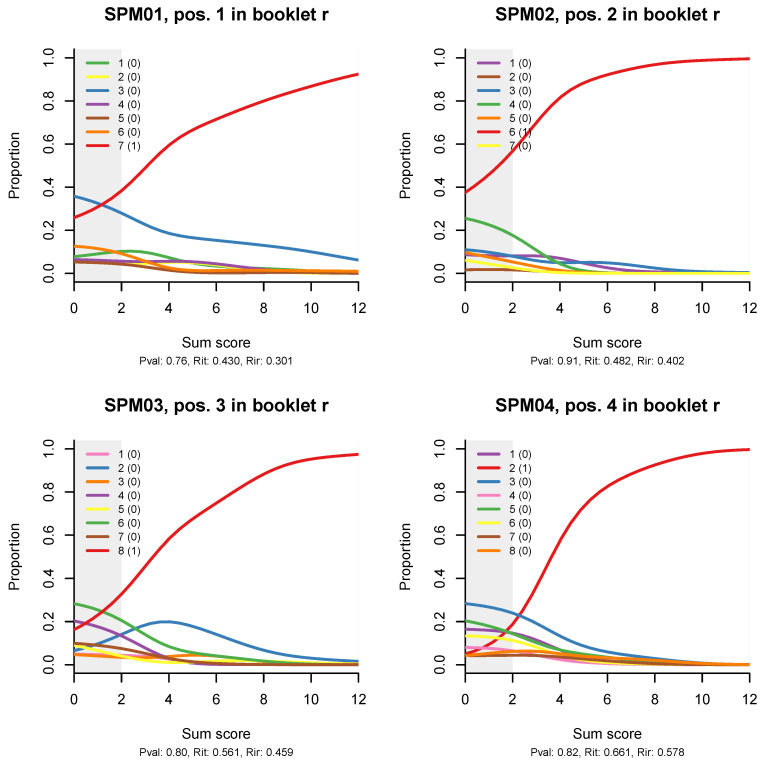

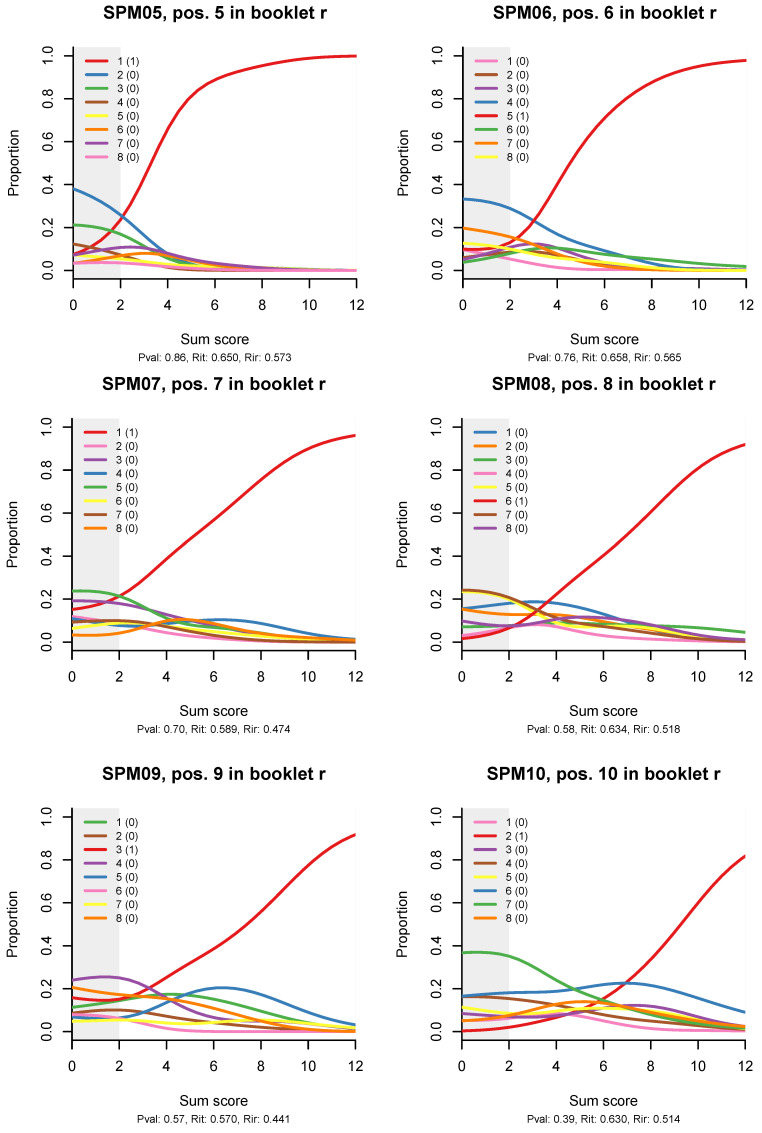

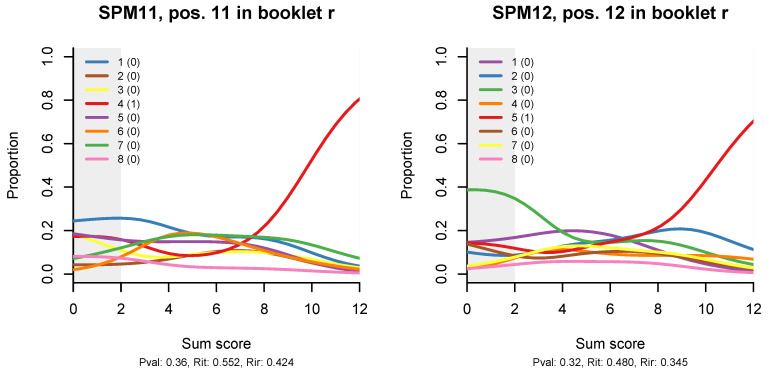
Non-parametric option-total regressions (distractor plots) for the twelve items in the SPM-LS test. The title of each plot shows the item label, in which booklet the item appears, and in what position. The legend shows the actual responses and the scores they will be given. Response alternatives that do not show up have not been chosen by any person.

Figure 4 shows distractor plots (non-parametric regressions of each response alternative to an item on the total test score), which are a less sophisticated but very detailed and useful diagnostic tool when an item appears spurious on the traditional CTT statistics and/or ITR plots. I have included the plots for all 12 items because an anonymous reviewer suggested that the reader would like to see them, and I completely agree; however, the idea is that, in practice, the CTT statistics and the ITR plots will help us narrow down the detailed diagnostic plots that we need to examine to the most problematic items.

Looking at the distractor plot for item SPM01, we observe that most of the seven distractors are not chosen at all, while one, represented with the blue line, is quite popular. When that happens, the item is effectively transformed into a coin tossing game. If this were a cognitive test, I would recommend rewriting the item. However, this is a matrix item, abstract and constructed in a principled manner, so the only explanation that comes to mind is that the test was given without a couple of items on which the examinees could train. For lack of these, the first item served as an exercise item.

Similar, but milder effects are observed on the ITR for items SPM09, SPM12, and possibly SPM11. The 3PL model (Appendix B) has larger pseudo-guessing parameters for these items. The distractor plots (Figure 4) show that all distractors are in use, but some of them tend to peak out in the middle, a bit like the middle category in a partial credit item. There might be some reason for this in the way Raven items are constructed.

There is more logic to guess when the item is difficult, especially if the stakes are high, but is this what is happening? Possibly. As one sharp-witted psychometrician told me once, the trouble with the research on guessing is that so much of it is guesswork. On the other hand, there must be ways to make guessing behaviour more explicit and include it in the rules of the game that we are playing. For example, one could have the subjects order the responses by their degree of confidence they are correct, or use a continuous response model as described in Verhelst (2019).

## 4. Discussion

To a pool of different analyses of the same dataset, I have contributed a specimen of the exploratory analysis we typically do when developing a cognitive test for assessment. My purpose was mainly to increase the diversity in professional perspectives, and to popularize some novel and useful diagnostic tools in our software.

While I use the Rasch model and the less popular interaction model, the focus is not on modelling, not even on the more traditional psychometric analysis of an intelligence test. Capitalizing on the fact that the models share the same scoring rule as the original test, the sum score, I use them to evaluate and support the scoring rule, and to highlight items that possibly go astray. I might have relied more heavily on the models in different circumstances: For example, if the test had more than one form (the Rasch model is useful for equating), or if I were interested in research, not in an instrument to assess persons.

The way in which I use the models explains why a paper that deals essentially with model fit does not treat model fit in the way typical of scientific research. I did not put forward any model to explain the data, in which case model fit would be an argument supporting my ideas. I did formulate a hypothesis or, rather, a guess (confirmed later) when I found out that a certain item did not follow my preferred model. In this case, model fit was about quality control more than about anything else.

I am certainly not original in pointing out that summative assessment, formative assessment, population surveys, methodological research and substantive research are sufficiently different to have not only distinct but sometimes even mutually exclusive criteria on what is desirable, appropriate, or admissible. This is fine as long as it is not forgotten and ignored.

In the final run, my story has three morals: (i) The way you should go “depends a good deal on where you want to get to” Carroll (1865), (ii) whatever the destination, always do exploratory analysis first, and (iii) in practical assessment, the model should follow from the scoring rule, not vice versa.

## Figures and Tables

**Table 1:** Selected item level CTT statistics for the SPM-LS data set.

Item	Facility	rit	rir
SPM01	0.76	0.43	0.30
SPM02	0.91	0.48	0.40
SPM03	0.80	0.56	0.46
SPM04	0.82	0.66	0.58
SPM05	0.86	0.65	0.57
SPM06	0.76	0.66	0.56
SPM07	0.70	0.59	0.47
SPM08	0.58	0.63	0.52
SPM09	0.57	0.57	0.44
SPM10	0.39	0.63	0.51
SPM11	0.36	0.55	0.42
SPM12	0.32	0.48	0.34

**Figure A1:**
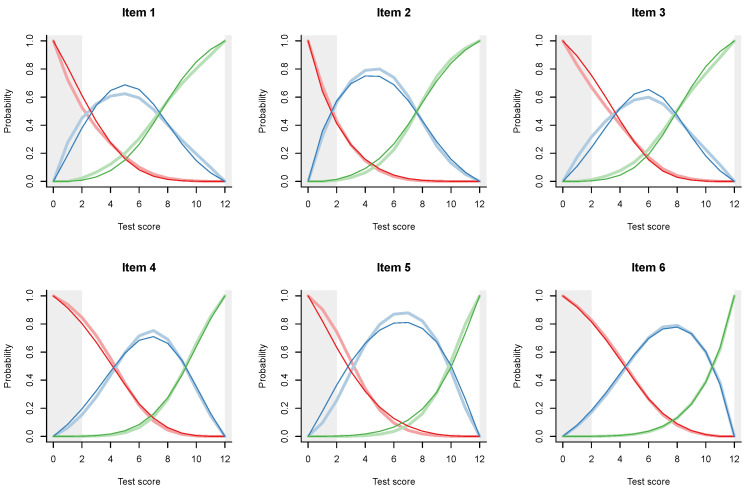
Category trace lines for partial credit items obtained by combining the original items SPM01 and SPM07 (Item 1), SPM02 and SPM08 (Item 2) etc. The partial credit model is shown with thinner and darker lines, and the polytomous IM with broader and lighter lines of the same hue.

**Figure A2:**
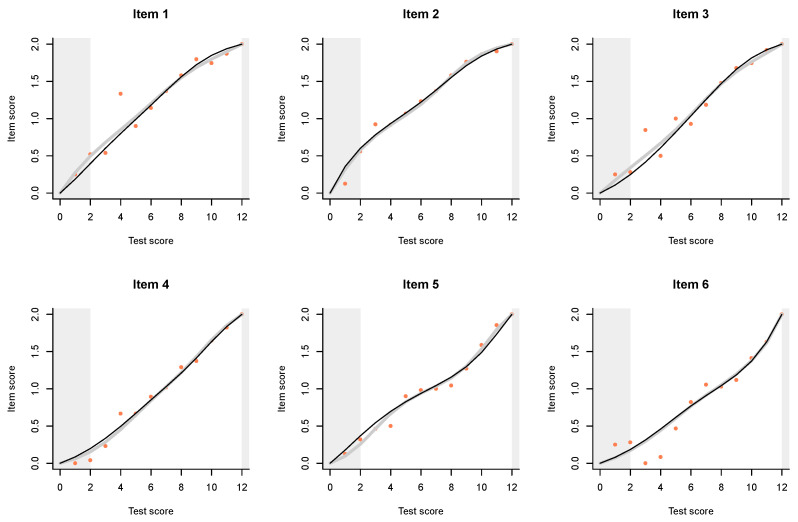
Item-total regressions for partial credit items obtained by combining the original items SPM01 and SPM07 (Item 1), SPM02 and SPM08 (Item 2) etc. Observed data is shown with pink dots, the PCM with thin black lines, and the interaction model with thick gray lines.

**Figure A3:**
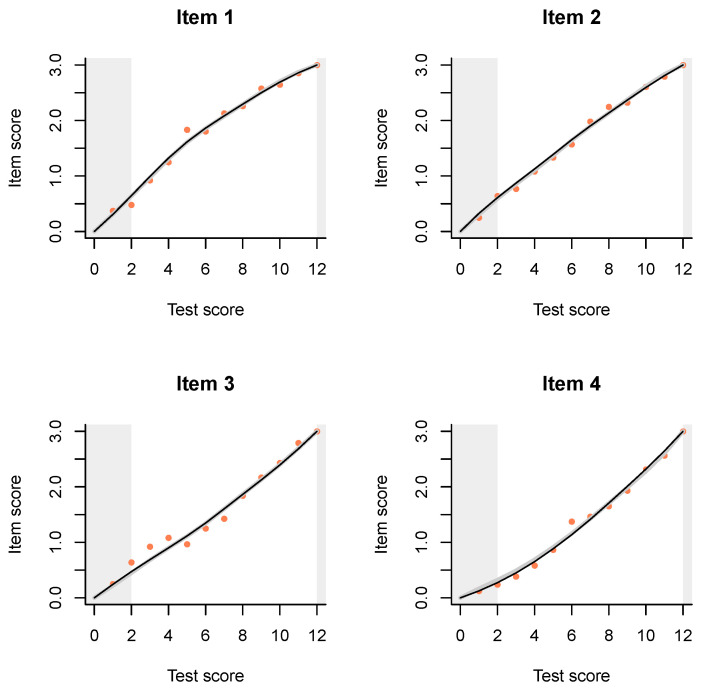
Item-total regressions for partial credit items obtained by combining triplets of items. Observed data is shown with pink dots, the PCM with thin black lines, and the interaction model with thick gray lines.

**Figure A4:**
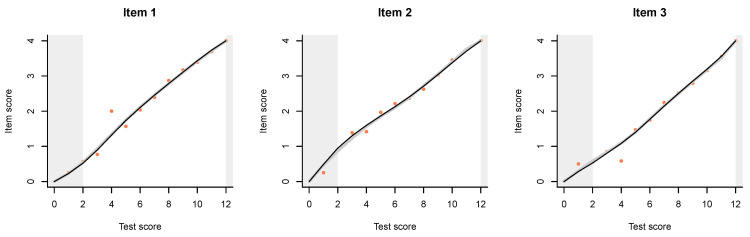
Item-total regressions for partial credit items obtained by combining quadruples of items. Observed data is shown with pink dots, the PCM with thin black lines, and the interaction model with thick gray lines.

**Figure A5:**
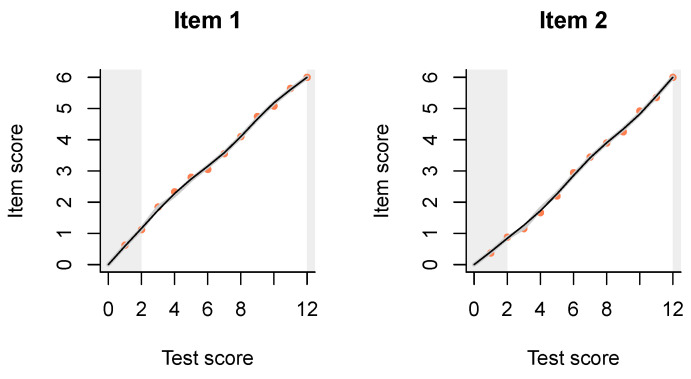
Item-total regressions for two subtests of six items each. Observed data is shown with pink dots, the PCM with thin black lines, and the interaction model with thick gray lines.

## Abbreviations

   The following abbreviations are used in this manuscript:

2PL	Two-parameter logistic (model)
3PL	Three-parameter logistic (model)
CTT	Classical test theory
IM	Interaction model
IRF	Item response function
IRT	Item response theory
ITR	Item-total regression
PCM	Partial credit model
SPM-LS	Standard Progressive Matrices (last series)

## Appendix A

This is a minimal script to perform all analyses in this paper. It does not cover the final formatting of the tables and graphs. Note that the original dataset was modified slightly by hand: column names were changed from STM1, STM2,...to STM01, STM02 etc.

library(dexter)	# load the dexter library
setwd(’~/WD/Raven’)	# set the work directory
keys = data.frame(	# data frame as required
item_id = sprintf(’SPM%02d’, 1:12),	# by keys_to_rules function
noptions = 8,	
key = c(7,6,8,2,1,5,1,6,3,2,4,5)	# (the correct responses)
)	
rules = keys_to_rules(keys)	# scoring rules as reqd by dexter
db = start_new_project(rules, ’raven.db’)	# data base from the rules
dat = read.csv(’dataset.csv’, head=TRUE)	# read in data...
add_booklet(db, dat, ’r’)	# ... and add to the data base
tia_tables(db)	# tables of CTT statistics
mo = fit_inter(db)	# fit the Rasch and the IM
plot(mo)	# produce all ITR plots
distractor_plot(db,’SPM01’)	# distractor plot for item SPM01

## Appendix B

I tried to estimate the 3PL model for the SPM-LS dataset with three different programs: The R package mirt Chalmers (2012), the R package ltm Rizopoulos (2006), and the long-time flagship in educational testing, BILOG-MG Zimowski et al. (1996). All options in the R packages were held at their defaults, and no priors were used in BILOG-MG to constrain any of the three parameters during estimation. The results are shown in Table A1.

We observe reasonably good agreement between mirt and BILOG-MG, while the ltm estimates deviate more. Interesting enough, the estimates of the pseudo-guessing parameter, *c*, seem to agree the most among the three programs. They are also logical: since the items are arranged by increasing difficulty, there is no logic to guess on the easy items, so the guessing parameter is zero or close to zero. For the two most difficult items, it is close to 1/8, but it is difficult to explain why people should want to guess more on item 9. Moreover, all three programs seem to struggle with the discrimination parameter of item 11, quite seriously in the case of ltm.

**Table A1:** Parameter estimates for the 3PL model obtained for the SPM-LS dataset with three different programs.

Item	Mirt Estimates			Ltm Estimates			BILOG-MG Estimates		
	*a*	*b*	*c*	*a*	*b*	*c*	*a*	*b*	*c*
SPM01	0.85	−1.55	0.00	0.87	−1.51	0.00	0.83	−1.57	0.00
SPM02	1.93	−1.82	0.00	2.00	−1.76	0.00	2.00	−1.80	0.00
SPM03	1.61	−1.24	0.00	1.66	−1.21	0.00	1.62	−1.24	0.00
SPM04	3.65	−1.01	0.00	4.31	−0.95	0.00	3.60	−1.02	0.00
SPM05	4.70	−1.11	0.00	5.59	−1.04	0.00	4.57	−1.13	0.00
SPM06	2.26	−0.89	0.00	2.36	−0.86	0.00	2.23	−0.91	0.00
SPM07	1.55	−0.75	0.02	1.57	−0.76	0.00	1.55	−0.75	0.02
SPM08	1.58	−0.29	0.00	1.62	−0.29	0.00	1.57	−0.28	0.00
SPM09	2.28	0.19	0.24	2.27	0.18	0.23	2.27	0.19	0.24
SPM10	2.09	0.35	0.00	2.15	0.34	0.00	1.88	0.39	0.00
SPM11	5.83	0.63	0.11	32.28	0.67	0.12	6.04	0.63	0.11
SPM12	3.39	0.90	0.14	3.25	0.88	0.14	3.35	0.91	0.14

I made another comparison using only BILOG-MG and playing with the available priors for constraining parameter estimates. Table A2 shows results without any priors at all (same as in Table A1); with a lognormal (0, 0.5) prior on the discrimination parameter, *a*, and no prior on *c*; and with a lognormal (0, 0.5) prior on *a* and a beta($20\times \frac{1}{8}+1$, $20\times \frac{7}{8}+1$) on *c* ($\frac{1}{8}$ and $\frac{7}{8}$ obtain from the fact that all items have 8 possible responses). The prior on *a* slightly alleviates the problem with the extremely high estimate while the prior on *c* simply invents guessing where we could not possibly have information on it: The less people have reason to guess, the more the estimate drifts towards $\frac{1}{8}$.

**Table A2:** Parameter estimates for the 3PL model obtained for the SPM-LS dataset with BILOG-MG and three different settings.

Item	Priors on *a* and *c*			Prior on *a*			No Prior		
	*a*	*b*	*c*	*a*	*b*	*c*	*a*	*b*	*c*
SPM01	0.90	−1.29	0.11	0.85	−1.53	0.00	0.83	−1.57	0.00
SPM02	1.93	−1.75	0.11	1.97	−1.80	0.00	2.00	−1.80	0.00
SPM03	1.65	−1.13	0.10	1.61	−1.24	0.00	1.62	−1.24	0.00
SPM04	3.23	−1.01	0.06	3.36	−1.03	0.00	3.60	−1.02	0.00
SPM05	3.85	−1.13	0.06	3.97	−1.15	0.00	4.57	−1.13	0.00
SPM06	2.34	−0.82	0.07	2.21	−0.90	0.00	2.23	−0.91	0.00
SPM07	1.64	−0.62	0.10	1.49	−0.80	0.00	1.55	−0.75	0.02
SPM08	1.67	−0.18	0.07	1.58	−0.29	0.00	1.57	−0.28	0.00
SPM09	1.79	0.05	0.16	1.91	0.10	0.19	2.27	0.19	0.24
SPM10	2.18	0.41	0.03	1.85	0.38	0.00	1.88	0.39	0.00
SPM11	3.97	0.64	0.10	3.98	0.63	0.10	6.04	0.63	0.11
SPM12	2.63	0.91	0.13	2.61	0.91	0.13	3.35	0.91	0.14

## Appendix C

One of the reviewers has asked me to add some references on model fit at test level. Taken sufficiently seriously, this is not quite as easy at it may seem. Flowing out of the theory of errors, CTT is very concerned with test reliability and validity. Classical texts on CTT Gulliksen (1950) have entire chapters devoted to, say, the effect of test length on test error, reliability, and validity. IRT has an indisputable contribution in focusing on the item and item fit, but it may have gone a bit too far, overlooking the proverbial forest for the sake of the trees.

For the more pragmatic outlook of this paper, an important reference concerned with the practical implications of model misfit is Sinharay and Haberman (2014). Reasoning similar to mine, but pertaining to differential item functioning (DIF) rather than item fit, is found in Chalmers et al. (2016).

In what follows, I will try to avoid the item level—test level dichotomy, and steal a peek in-between. Our software, dexter Maris et al. (2019), has a handy function, fit_domains(), for the analysis of subtests within the test. The function transforms the items belonging to each subtest, or domain, into one large partial credit item. Such ‘polytomisation’, as discussed by Verhelst and Verstralen (2008), is a simple and efficient way to deal with testlets. The formal, constructed, and homogeneous nature of the SPM-LS test makes it a good candidate for some further experimentation. Note that I am not proposing a new method—I am just being curious.

I start by combining item 1, intended to be the easiest, with item 7, of medium difficulty. Item 2 will be combined with item 8, item 3 with item 9, and so on. We end up with 6 partial credit items combining an easier item with a more difficult one or, if you wish, six testlets or subtests made to be as parallel as possible. Their category trace lines are shown on Figure A1.

We can also examine the ITR (Figure A2), which are comparable to the ITR for the original test items; the item score on the *y* axis is also the ‘test score’ of the six subtests. We observe better item fit and a closer correspondence between the regressions predicted by the two models.

The next step will be to combine triplets of items: 1, 5, and 9; 2, 6, and 10, etc. Perhaps not surprisingly, the two models and the data come even closer (Figure A3).

Quite predictably, the next step is to combine quadruples of items, and the result is even better fit (Figure A4).

Finally, we have two subtests of six items each, one consisting of the odd-numbered items, and the other of the even-numbered items in the original test (Figure A5). This parcours is the closest approximation to item fit at test level from an IRT perspective that I can produce as of now.
